# Needs satisfaction and Online Self-Regulated Learning among chinese undergraduates

**DOI:** 10.1371/journal.pone.0321781

**Published:** 2025-04-28

**Authors:** Xiaohua Zhou, Ching-Sing Chai, Morris Siu-Yung Jong, Huiya Feng

**Affiliations:** 1 Teaching and Learning Centre, Lingnan University, Hong Kong, Hong Kong SAR; 2 Department of Curriculum and Instruction, The Chinese University of Hong Kong, Hong Kong, Hong Kong SAR; 3 Centre for Learning Sciences and Technologies, The Chinese University of Hong Kong, Hong Kong SAR; 4 Academic Affair Office, Guangzhou City University of Technology, Guangzhou, China; The University of Auckland, NEW ZEALAND

## Abstract

The increasing popularity of online courses has highlighted the importance of online self-regulated learning (OSRL). However, its use among undergraduates remains challenging. Based on self-determination theory, this study examined whether satisfying three basic psychological needs (i.e., autonomy, competence, and relatedness) could foster the engagement of Chinese undergraduates (N = 381) in OSRL. Data were collected from an online questionnaire, which included a revised Online Self-Regulated Learning Questionnaire, a scale to measure need satisfaction, and demographic information items. Descriptive analyses, confirmatory factor analyses and structural equation modeling were conducted. The results revealed that overall need satisfaction was positively associated with the use of OSRL strategies. Specifically, satisfying autonomy was positively associated with the use of resource management strategies. Feeling competent was positively associated with most OSRL strategies, except for time management and help seeking. A sense of relatedness was positively associated with self-evaluation, task strategies, and help seeking. These findings underscore the unique role of each need in promoting OSRL. Therefore, designers and instructors of online courses should cater to the specific needs of undergraduates to enhance their use of targeted OSRL strategy(ies).

## Introduction

Online courses have become increasingly prevalent in higher education [1]. Universities that formerly offered only on-campus courses are increasingly switching to online courses to meet increasing student demand [2], and this was especially the case during the COVID-19 pandemic [3].

However, online self-regulated learning (OSRL) may be a problem among university students because of the increased onus on them to manage their own learning in online classes [4,5]. Schwam et al. [6] reported that, based on profile analyses, a majority of university students exhibited relatively low levels of OSRL. Furthermore, according to a previous study, university students tend to rely on familiar learning strategies rather than adopting more efficient ones [7]. OSRL was reported as the most pressing challenge faced by university students in online learning during COVID-19 (Kohnke et al., 2021). Particularly, researchers highlighted an urgent need for more work to be done in fostering university students’ metacognitive strategies of OSRL, e.g., setting learning goals, reflecting and evaluating learning [8,9]. Additionally, the lack of ability to manage online learning was significantly correlated with a decrease in study time during COVID-19 among Chinese university students [10].

According to self-determination theory (SDT) [11,12], people can be energised to self-regulate when their basic psychological needs (i.e., autonomy, competence, and relatedness) are satisfied. Satisfying these three needs leads to a sense of vitality (the experience of feeling alive, vigorous, and energetic) and full functioning (not only free of psychopathology but also capable of harnessing energy to engage in activities that one values [11]). This state of vitality and full functioning may facilitate self-regulated learning. Empirical studies conducted in face-to-face settings have supported the positive association between need satisfaction and self-regulated learning [13,14]. However, further studies are needed to determine whether this association also applies to online learning settings. Moreover, exploring which specific needs are associated with which strategies is crucial. Therefore, the present study investigates the associations between basic psychological need satisfaction and OSRL strategies among undergraduates. Hereinafter, basic psychological needs are referred to simply as needs.

## Literature review

### Online self-regulated learning

According to Boekaerts and Cascallar [15], self-regulation refers to “multicomponent, iterative, self-steering processes that target one’s own cognitions, feelings, and actions, as well as features of the environment for modulation in the service of one’s own goals.” (p. 199). Azevedo et al. [16]stated that “self-regulated learners are generally characterised as individuals who actively and efficiently manage their learning through monitoring and strategy use.” (p. 173). In this study, OSRL refers to the process of self-regulation, such as proactive goal setting, adjusting, and adapting, in online learning settings.

The OSRL strategies that are commonly measured in empirical studies include metacognitive strategies, which involve students’ awareness of their learning process, as well as their ability to set goals and monitor their learning process (e.g., goal setting, self-evaluation); cognitive strategies, which pertain to students’ management of cognitive processes to perform learning tasks (e.g., task strategies); and resource management strategies, which involve students using the resources around them to achieve their goals, for example, time management, environment structuring, and help seeking [17–19].

The use of OSRL strategies can be influenced by a variety of internal (i.e., student-specific) and external (i.e., environmental) factors. Internal factors, including university students’ academic self-efficacy [20], beliefs about knowledge and learning [21], emotions [22], personalities [23], and prior online learning [24], were found to be associated with their use of OSRL strategies. External factors, such as pedagogical interventions (e.g., prompting and feedback [25]) and tools (e.g., metacognitive scaffolding tools) [26], were reported to be effective in encouraging the use of OSRL strategies. The present study focused on an internal condition, namely need satisfaction, which is key to self-regulation [27] and has not been sufficiently examined in the context of OSRL.

### Self-determination theory and online self-regulated learning

According to the SDT, autonomy, competence, and relatedness are basic psychological needs [11]. Autonomy refers to the need for freedom or choice, competence refers to the feeling of being competent and having a sense of mastery, and relatedness refers to a sense of feeling connected to other people [11]. Satisfying these three needs leads to subjective vitality, full functioning, enhanced psychological well-being, and personal adjustments [11,26]. Although the SDT has long been applied in education contexts, the researchers who proposed this theory have recently encouraged scholars to more thoroughly investigate how educational technologies motivate engagement and learning through need satisfaction [12].

In face-to-face learning settings, Sava et al. [14] observed that perceived autonomy, perceived competence, and teacher support were correlated with SRL among university students. Mukhtar [14] reported that perceived competence encouraged the use of metacognitive strategies and resource management strategies among university students. The shift in the mode of instruction from face-to-face to online promotes anywhere, anytime learning and involves different competencies and ways to relate with course mates, so there should be substantive variations in self-regulation strategies that warrant dedicated research. To be more specific, first, in terms of resource management, the online learning requires different approaches to managing time, the learning environment, and seeking help through online communication. For example, time management has been consistently reported as a challenge in online learning [4,10,28]. Moreover, managing the learning environment (e.g., coping with distractions at home, selecting study areas, and setting a study schedule) is one of the most difficult challenges for online learning during COVID-19 [28]. Second, cognitive strategies may differ between face-to-face and online learning settings. For instance, students multitask more in online courses than in face-to-face courses, which means they pay less attention to the online course content [29]. Additionally, the ways students take notes and engage in discussions vary between online and face-to-face courses. For example, in online courses, students may capture screens, speed up video playback, or use emojis and reactions to respond to peers and instructors – practices that are less common in face-to-face courses. Third, metacognitive strategies may need to be used more frequently in online learning settings, as students need to take on more responsibility for monitoring their progress, reflecting on their learning, and adjusting their goals.

Apart from differences in these strategies, the association between needs and strategies could also differ. For example, students enjoying more autonomy may not lead to changes the study schedule in face-to-face classrooms, where instructors typically control the learning schedule and pace. However, when students have more autonomy in online learning settings, the gap between students’ actual learning pace and their desired pace may become larger than in face-to-face environments. In other word, when students enjoy more autonomy, time management become more important in online learning environment, especially in asynchronous online courses. In sum, the use of OSRL strategies and the links between these strategies and the needs could differ from those in face-to-face environment.

In online learning settings, satisfying students’ needs was reported to enhance students’ behavioral, emotional, cognitive, and agentic engagement [30]; improve perceived knowledge transfer [31]; foster perceived usefulness, perceived enjoyment, and continued intention to OSRL [32]; increase intention to continue with online learning [33]; increase learner satisfaction [34]; lead to more active learning behavior and less passive learning behavior [35]; and reduce academic procrastination [36]. However, the association between need satisfaction and OSRL, which is an important variable in online learning, has been underexplored.

One study that was closely related to the present study is Holzer et al.’s study [37], which examined the association between need satisfaction and psychological well-being by considering goal setting and planning as moderating variables. The present study differs from Holzer et al. [37] in several respects. First, the present study focused on the association between need satisfaction and OSRL, whereas Holzer et al. [37] focused on the association between need satisfaction and psychological well-being. Second, the present study focused on six OSRL strategies, whereas Holzer et al. [37] involved only goal setting and planning. Third, in the examination of the moderation effect of SRL on the association between need satisfaction and psychological well-being, Holzer et al. [37] examined only autonomy and competence as moderators, but without relatedness. By contrast, the present study included relatedness because it was found to have a significant positive effect on OSRL [38] among Chinese university students.

More recent studies [39,40] reported moderating and mediating roles of need satisfaction between artificial intelligence and SRL among K-12 students, based on SDT. However, the role of need satisfaction in online learning for university students requires further clarification.

### Satisfaction of each psychological need and online self-regulated learning

Apart from the effects of overall need satisfaction on OSRL, this study also aims to examine the association between satisfaction of each psychological need and OSRL. According to SDT, the three needs, i.e., autonomy, competence, and relatedness, are three different needs and tap into different motivational and socioemotional systems. Therefore, they may influence OSRL differently. For instance, when the need for relatedness is fulfilled, students tend to use socially supported strategies, such as seeking help from others. Durksen et al. [41] have revealed that relatedness is distinct from autonomy and competence in the context of MOOC (Massive Open Online Course) learning and encourage future research on this topic. Although autonomy and competence are more closely related to each other [41], they may exert different effects on OSRL. For example, individuals who feels more competent may set higher goals, whereas satisfying the need for autonomy may not have the same effect.

The varying effects of each need on OSRL strategies are evident in existing studies, which report these effects inconsistently. First, some studies have reported that the satisfaction of each need can predict online learning outcomes, with competence being the strongest predictor, followed by relatedness and autonomy. Chen and Adesope [42] discovered a significant association between the satisfaction of each need and overall satisfaction with online general education courses among university students [competence (ß = 0.37)> relatedness (ß = 0.29)> autonomy (ß = 0.24)]. Fang et al. [43] revealed that competence was the strongest predictor of learning engagement, followed by relatedness and then autonomy among MOOC learners. Second, Holzer et al. [37] revealed that competence was the sole positive predictor of psychological well-being (operationalized as positive emotion and intrinsic learning motivation), whereas satisfying autonomy and relatedness did not necessarily predict psychological well-being in the context of online learning among university students. The present study extends these empirical findings by determining the associations of each need with OSRL strategies.

In summary, whether need satisfaction is positively associated with OSRL and the associations between each need and each OSRL strategy remain to be addressed. We have learned that need satisfaction is associated with SRL. However, due to the differences between face-to-face and online learning settings, it is necessary to examine this association in an online learning setting. Moreover, each need may play a different role in shaping OSRL, which has been inconsistently reported and requires further examination. Moreover, from a practical point of view, if online course instructors understand not only whether satisfying students’ needs can help enhance their OSRL but also which needs play a more important role or affect specific OSRL strategies, their course design and teaching practices to enhance students’ OSRL could become more targeted and effective.

### The present study

The present study aims to (a) examine the associations between overall need satisfaction and the use of OSRL strategies and (b) examine the specific associations between the satisfaction of each need (i.e., autonomy, competence, and relatedness) and the adoption of OSRL strategies among undergraduates. OSRL includes six strategies within three dimensions: metacognitive strategies (i.e., goal setting and self-evaluation), cognitive strategies (i.e., task strategies), and resource management strategies (i.e., time management, environment structuring, and help seeking).

## Method

### Context and participants

The research protocol was approved by the Survey and Behavioral Research Ethics Committee in The Chinese University of Hong Kong. The written consent were obtained from participants. The study was conducted among undergraduates who had enrolled in online general education courses during the 2021–2022 fall semester at a university in South China. This university offered a total of 21 online general education courses to undergraduates in all disciplines through two widely used online course platforms in China (12 courses on http://erya.mooc.chaoxing.com and nine courses on https://www.zhihuishu.com). The courses covered various topics, including public health (e.g., Infectious Diseases), art appreciation (e.g., World Classic Arts Appreciation), humanities (e.g., Eastern and Western Philosophies), and innovation and entrepreneurship (e.g., From Idea to Business). The courses comprised prerecorded video lectures, reading materials, quizzes, a discussion forum, and a final examination. Each course carried two credits. The courses had no involvement of an instructor or teaching assistant in the learning process, and undergraduates were required to complete their courses independently by the end of November 2021. In other words, the courses were conducted fully online in an asynchronous manner. Therefore, the findings may not be generalizable to blended courses or synchronous online courses.

In this study, students were recruited through convenience sampling for an online questionnaire survey. After the course exam period, student helpers distributed the survey link to their classmates. The link was open for one week from December 12 to 19, 2021. Prior to starting the survey, students received information about the research goals, requested time for completing the questionnaire, and data confidentiality. Those who agreed to participate in the study and provided written consent could proceed to complete the questionnaire. A total of 381 undergraduates completed the survey. The average age of the participants was 20.19 (SD = 1.08), and of the total participants, 192 were female (50.39%). The participants were distributed across different academic years, with the majority being in year 2 (n = 216, 56.69%), followed by year 3 (n = 131, 34.38%) and year 4 (n = 34, 8.92%). They came from 12 faculties, with the three major ones being the Faculty of Foreign Languages (n = 81, 21.26%), Faculty of Management (n = 65, 17.06%), and Faculty of Electronics and Information Engineering (n = 47, 12.34%).

## Measures

### Online self-regulated learning

To measure participants’ OSRL, a revised version of the Online Self-Regulated Learning Questionnaire (OSLQ) [17] which originally comprised 24 items and 6 dimensions (goal setting, self-evaluation, task strategies, time management, environment structuring, and help seeking), was used. Because learning is highly contextual and OSLQ items did not accurately fit the Chinese context in a previous study [38], seven items of the OSLQ were substituted with corresponding items from the Self-regulated Online Learning Questionnaire Revised (SOL-Q-R) [19]. For example, the item, “Although we have to attend daily classes, I still try to distribute my studying time evenly across days.” in the OSLQ was substituted with “I allocate study time for this online course.” in the SOL-Q-R, because the participants in our study did not have to attend classes every day. The revised questionnaire items were then reviewed by two experts with university teaching experience to ensure content validity. Subsequently, the items were pilot tested among 93 Chinese university students who were not part of the main study. Based on the review and pilot test results, one goal setting-item from the OSLQ was replaced with a goal-setting item from the SOL-Q-R, and two items were discarded from the final questionnaire. The final questionnaire comprised 22 items. The six subdimensions were identical to those of the OSLQ. The dimensions and example items are presented in Table 1. The Cronbach’s alpha values for the original subscales of the OSLQ ranged from 0.67 to 0.90 [16]. In the present study, they ranged from 0.79 to 0.90, indicating good internal consistency.

**Table 1:** Measures.

Dimension	No. of items	Example item
Online self-regulated learning		
Goal setting	4	I set standards for my assignments in this online course.
Task strategies	4	I try to take more thorough notes for this online courses because notes are even more important for learning online than in a regular classroom.
Time management	3	I allocate study time for this online course.
Environment structuring	3	I choose the location where I study to avoid too much distractions.
Help seeking	4	When I do not fully understand something, I ask other course mates in this online course for ideas.
Self-evaluation	4	I summarize what I have learned in this online course to examine my understanding of what I have learned.
Need satisfaction		
Autonomy	4	I feel free to make my own decisions in this online course.
Competence	4	I think I am pretty good at learning this online course.
Relatedness	4	I feel close to my course mates in this online course.

### Need satisfaction

To assess the level of need satisfaction of the participants, we adopted 12 questionnaire items (four for each need) from Chiu’s study [30]. The items were slightly modified to fit the specific context of the present study and tested on the aforementioned group of 93 Chinese university students. For example, “I enjoy interacting with my classmates” was modified to “I enjoy interacting with my course mates in this online course.” Example items are listed in Table 1.

All the questionnaire items used to measure OSRL and need satisfaction were scored on a 5-point Likert scale, from 1 = *strongly disagree* to 5 = *strongly agree*. The items were translated into Chinese using a back translation approach.

### Data analyses

**Fig 1:**
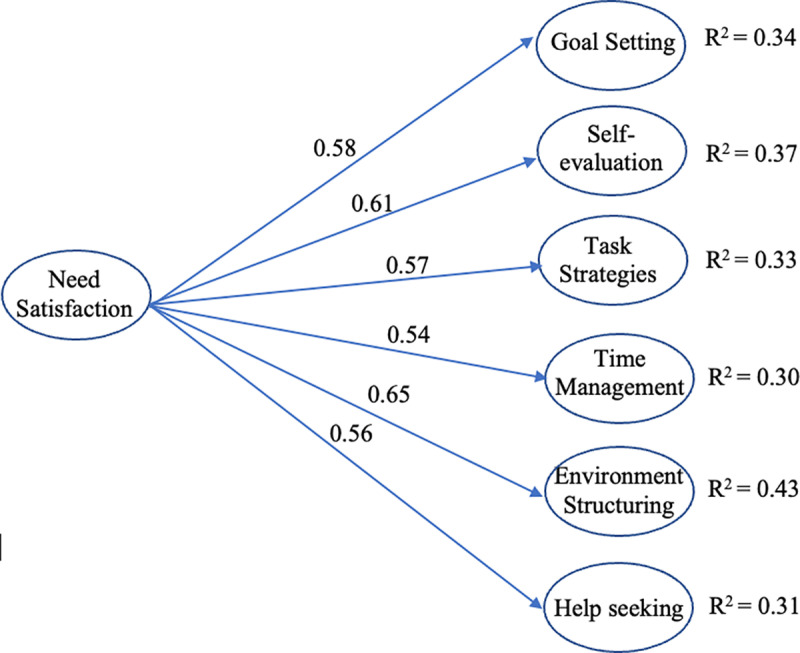
Path coefficients of M1 (second-order model with need satisfaction as the exogenous variable). *Note.* All path coefficients are significant at *p* <.001 level.

**Fig 2:**
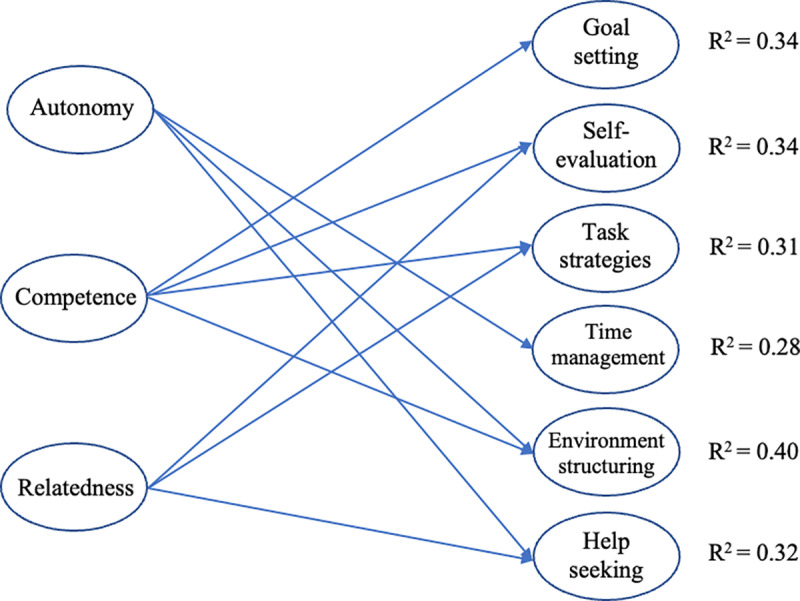
M2 (first-order model considering the three needs as the exogenous variables). *Note.* Only significant paths are shown.

We first examined the reliability and validity of all the scales, calculated descriptive statistics, and then used structural equation modeling (SEM) to test the proposed models. Two models were tested: M1 and M2. M1 employed the second-order factor (i.e., need satisfaction) as the exogenous variable (Fig 1), whereas M2 employed only the first-order factors (i.e., autonomy, competence, and relatedness) as the exogenous variables (Fig 2). M1 served as the basis for M2. After establishing the positive association between need satisfaction and OSRL, we further examined the unique contributions of each need to different OSRL strategies. Because participants took part in the online courses from two platforms, platform type was treated as a confounding variable (platform A = 1, platform B = 0) in both the models.

## Results

### Scale reliability and descriptive statistics

The subscales were reliable. Specifically, all subscales related to OSRL and need satisfaction had acceptable internal consistencies, with Cronbach alphas ranging from 0.75 to 0.90 and with composite reliability of each subscale being > 0.70 (Table 2) [44].

**Table 2:** Descriptive statistics.

	GS	SE	TS	TM	ES	HS	AT	CP	RL	NS
Mean	3.61	3.65	3.48	3.62	3.78	3.65	3.89	3.66	3.61	3.70
SD	0.91	0.82	0.86	0.87	0.81	0.84	0.68	0.72	0.84	0.64
Cronbach’s α	0.90	0.86	0.84	0.83	0.79	0.85	0.75	0.83	0.88	0.90
CR	0.90	0.86	0.85	0.83	0.79	0.85	0.75	0.83	0.88	0.97

*Note.* GS = goal setting, SE = self-evaluation, TS = task strategies, TM = time management, ES = environment structuring, HS = help seeking, AT = autonomy, CP = competence, RL = relatedness, NS = need satisfaction, SD = Standard Deviation, CR = Composite reliability.

Table 2 presents the descriptive results of the measured variables. Regarding OSRL strategies, participants rated environment structuring the highest and task strategies the lowest. Regarding need satisfaction, participants scored autonomy the highest and relatedness the lowest. The skewness of all the observed variables ranged from |0.28| to |0.90| (<|3|), and kurtosis ranged from |0.03| to |1.23| (<|8|), indicating that the data were normally distributed [45]. The six OSRL strategies and the three needs were significantly positively correlated, with coefficients ranging from 0.34 to 0.49 (bold in Table 3).

**Table 3:** Correlations among key variables.

	GS	SE	TS	TM	ES	HS	AT	CP	RL
GS	(0.83)								
SE	0.65	(0.78)							
TS	0.69	0.65	(0.76)						
TM	0.71	0.63	0.66	(0.79)					
ES	0.59	0.69	0.63	0.69	(0.74)				
HS	0.61	0.78	0.65	0.61	0.72	(0.77)			
AT	**0.35**	**0.40**	**0.35**	**0.40**	**0.46**	**0.42**	(0.71)		
CP	**0.49**	**0.48**	**0.44**	**0.41**	**0.48**	**0.40**	0.62	(0.74)	
RL	**0.38**	**0.44**	**0.42**	**0.34**	**0.38**	**0.43**	0.50	0.58	(0.80)

*Note.* All correlations are significant at the 0.01 level. Items on the diagonal are the square roots of the average variance extracted (AVE); off-diagonal elements are the correlation estimates. GS = goal setting, SE = self-evaluation, TS = task strategies, TM = time management, ES = environment structuring, HS = help seeking, AT = autonomy, CP = competence, RL = relatedness, NS = need satisfaction. The correlations between OSRL subscales and SDT subscales are in bold.

### Structural equation model

Before conducting the SEM analyses, a confirmatory factor analysis (CFA) was performed for OSRL and need satisfaction scales. For the OSRL scale, the CFA results demonstrated a good model fit [*x*^2^ = 424.91, df = 194, *p* <.001, *x*^2^/df = 2.19 (<5), CFI = 0.96 (>0.90), TLI = 0.95 (>0.90), RMSEA = 0.06 (<0.08), SRMR = 0.04 (<0.05)]. The factor loadings of each item ranged from 0.72 to 0.87. For the need satisfaction scale, one item was discarded from the autonomy subscale because its factor loading was weak. The CFA results for the remaining 11 items indicated good model fit [*x*^2^ = 66.24, df = 41, *p* <.001, *x*^2^/df = 1.62 (<5), CFI = 0.99 (>0.90), TLI = 0.98 (>0.90), RMSEA = 0.04 (<0.08), SRMR = 0.03 (<0.05)]. The factor loadings of each item ranged from 0.69 to 0.83. Moreover, the average variance extracted (AVE) of each subscale was >0.5, and the square root of the AVE was generally greater than the inter-construct correlations (Table 3), indicating acceptable convergent and discriminant validity [44]. Overall, the CFA results and the AVE values indicated that the measurements were good for conducting SEM analyses.

Two models, M1 and M2, were evaluated in the present study. Both models had acceptable fit (Table 4). The findings from M1 revealed that need satisfaction was positively related to OSRL strategies, with ß ranging from 0.54 to 0.65. Moreover, need satisfaction explained 30%–43% of the variance for each OSRL strategy (Fig 1).

**Table 4:** Model fit indices for the SEMs.

	M1 (2^nd^ order)	M2 (1^st^ order)	Model fit indices criteria (Hair et al., 2010)
chi-square	891.02	829.92	
p	<0.000	<0.000	
df	497	483	
chi-square/df	1.97	1.72	<5
CFI	0.95	0.96	> 0.90
TLI	0.94	0.95	> 0.90
RMSEA	0.05	0.04	< 0.08
SRMR	0.04	0.04	< 0.05

The results from M2 indicated that each need had a distinct association with the use of OSRL strategies (Fig 2 and Table 5). Specifically, students’ perceived autonomy was positively related to resource management strategies (i.e., time management, environment structuring, and help seeking), with ß ranging from 0.30 to 0.39. Perceived competence was positively associated with metacognitive strategies (i.e., goal setting and self-evaluation), cognitive strategy (i.e., task strategies), and only one resource management strategy (i.e., environment structuring), with ß ranging from 0.30 to 0.58. Perceived relatedness was positively correlated with one metacognitive strategy (i.e., self-evaluation), cognitive strategies (i.e., task strategies), and one resource management strategy (i.e., help seeking), with ß ranging from 0.22 to 0.25. Each need explained 28%–40% of the variance for each OSRL strategy.

**Table 5:** Path coefficients from satisfaction of each need and overall need satisfaction to six OSRL strategies.

	GS	SE	TS	TM	ES	HS
AT	-0.09	0.08	0.07	0.32*	0.30*	0.39**
CP	0.58***	0.35**	0.30*	0.18	0.31*	-0.02
RL	0.10	0.22**	0.24**	0.08	0.07	0.25**
NS	0.58***	0.61***	0.57***	0.54***	0.65***	0.56***

*Note.* *** *p* <.001, ** *p* <.01, * *p* <.05.

GS = goal setting, SE = self-evaluation, TS = task strategies, TM = time management, ES = environment structuring, HS = help seeking, AT = autonomy, CP = competence, RL = relatedness, NS = Need Satisfaction.

## Discussion

This study examined how the satisfaction of needs in general and the satisfaction of each need (i.e., autonomy, competence, and relatedness) are associated with the use of OSRL strategies. In general, the results highlighted the importance of satisfying undergraduates’ psychological needs during online learning. Students engaged in online learning are not only students seeking knowledge or skills but also individuals with basic psychological needs that must be fulfilled to maximize the benefits of online learning. Satisfying these needs could enhance the students’ OSRL. This finding is consistent with studies on need satisfaction predicting SRL in face-to-face learning settings [13,14]. More importantly, this study highlighted the distinct roles of each need in enhancing the use of OSRL strategies.

### Autonomy and the use of OSRL strategies

Autonomy was positively associated with resource management strategies (i.e., time management, environment structuring, and help seeking). That is, when students experienced a freedom of choice in their online learning, they tended to adopt resource management strategies to optimize their learning experiences. One possible reason for the positive associations between autonomy and time management as well as environment is the flexibility in study schedule offered by asynchronous online courses and the freedom for undergraduates to select learning environment (e.g., library, dormitory, home) in this study. This is consistent with the previous finding that, in asynchronous online learning, university students use time management and environment structuring strategies more frequently than in synchronous learning [28]. However, the positive association between autonomy and help-seeking requires further examination. It is possible that a third variable mediates the relationship between the two. For example, in an asynchronous online course, students enjoy greater freedom and autonomy but lose the opportunity to receive immediate instruction and feedback. This may lead to increased confusion or problems, which, in turn, could result in more use of help-seeking strategy.

Satisfying the need for autonomy was not associated with changes in the use of metacognitive or cognitive strategies. This suggests that students’ engagement in metacognitive and cognitive strategies may not be significantly influenced by the degree of freedom they experience. Students may adopt goal setting, self-evaluation, and task strategies in the same manner irrespective of whether they enjoy more or less freedom of choices. One possible explanation is that undergraduates may not be aware of the range of metacognitive and cognitive strategies available to them. They might rely on familiar and habitual learning strategies, such as quizzes, which they have used throughout their educational journey [7]. To address this limitation, in addition to providing students with more choices in their learning experiences, they can be introduced to more empirically supported metacognitive and cognitive strategies for OSRL. This could be achieved through explicit (e.g., workshops on OSRL strategies for first-year undergraduates) or implicit (e.g., tools for metacognitive scaffolding) [25] means.

In summary, the study highlights that when undergraduates perceived greater autonomy and freedom of choice in their online learning, they tended to focus on managing their resources rather than employing metacognitive or cognitive strategies. More training and scaffolding on metacognitive and cognitive strategies may be needed for undergraduates.

### Competence and the use of OSRL strategies

Satisfaction of competence was demonstrated to be positively associated with four out of six OSRL strategies: goal setting, self-evaluation, task strategies, and environment structuring. Students who felt competent in their online learning were more inclined to use these four strategies than students who did not feel competent. It is generally consistent with previous research, which reports that students who perceive themselves as highly competent coped better and used more OSRL strategies compared to students who perceived themselves as less competent [46], and the research that reported an association between satisfaction of competence and the use of learning strategies [47]. This is aligned with the SDT, which posits that the satisfaction of the three basic psychological needs can enhance students’ intrinsic motivation [12]. Students who are intrinsically motivated are more likely to employ learning strategies to achieve their goals than those who are not. Another possible reason is that when undergraduates felt competent in their ability to successfully learn online courses, they were more inclined to set higher expectations. That is, their goal extended beyond merely completing the course. Thus, they employed more metacognitive and cognitive strategies. This effect cannot be achieved by satisfying students’ need for autonomy, as mentioned above. From this perspective, to increase the use of metacognitive and cognitive strategies, online course instructors should help undergraduates feel a greater sense of competence instead of merely offering more choices.

Although this finding regarding the positive role of satisfying competence in OSRL is generally aligned with the SDT and previous studies, the specific relationship between satisfying competence and each OSRL strategy is only partially consistent with those in prior research. The positive association between perceived competence and metacognitive strategies (i.e., goal setting and self-evaluation) is consistent with those in previous studies [13,14]. Similarly, the positive association between competence and resource management strategies is consistent with the findings of Sava et al. [13]. However, the positive association between perceived competence and cognitive strategies does not align with the findings of other studies [12,13]. This inconsistency may be attributed to differences between the measurements used in these studies and the present study. Mukhtar [13] and Sava et al. [14] used the cognitive strategies subscale of the Motivated Strategies for Learning Questionnaire (MSLQ) [48], which was originally developed for face-to-face learning and measured four strategies: rehearsal, elaboration, organization, and critical thinking, using 19 items. In contrast, the present study used the task strategies subscale of the OSLQ, which was developed for online learning, to measure how students complete a task strategically, with four items. Therefore, the results regarding cognitive strategies are not comparable between the present study and previous studies [13,14]. Additional studies are warranted to replicate our findings in the future.

However, feeling competent was not associated with time management. One possible explanation for this finding is that undergraduates who perceive themselves to be competent may prioritize metacognitive and cognitive strategies over managing their study time. They focused more on how to complete tasks strategically and monitor their learning process rather than on allocating study time. One reason may be the students who feel competent would not worry too much about study time. Additionally, satisfying the need for competence was not associated with help seeking. This finding is consistent with the study of Credé and Phillips [49], which reported that high-performing college students less frequently engaged in the help seeking strategy than their moderately successful peers.

In summary, satisfying students’ competence could encourage them to use more goal setting, self-evaluation, task strategies, and environment structuring. To foster a sense of competence and more effective use of various OSRL strategies, online course instructors and designers can provide students with clear instruction and guidance [50], challenges that match students’ skills [51], and positive feedback [52].

### Relatedness and the use of OSRL strategies

Relatedness was positively associated with three OSRL strategies: self-evaluation, help seeking, and task strategies. The positive association between relatedness and self-evaluation may be attributed to the fact that individuals tend to engage more in social comparison with those they feel close to [53]. Students who feel connected to their peers are more inclined to interact and share their learning progress, which may trigger their self-evaluation. By contrast, socially disconnected students are less likely to communicate about their learning progress, and thus, they may engage less in self-evaluation. The positive association between perceived relatedness and help seeking can be explained by the social dimension of this need. It could be easier to ask for help from someone to whom an undergraduate feels connected. Particularly, interpersonal trust was found to play a crucial role in knowledge-seeking among Chinese students [54]. The positive association between relatedness and task-strategies may be because when students feel connected to peers in an online course, they tend to be more engaged in that online course and thus employ more task strategies [55]. These results emphasize the importance of building a strong learning community in online courses to promote self-evaluation, task strategies, and help seeking among undergraduates.

Relatedness was not associated with time management or environment structuring. One possible explanation for this lack of association is that these two strategies are more personal than the others. Unlike k-12 students, undergraduates have highly individualized study schedules and living arrangements. Similarly, relatedness was not associated with goal setting, which may be attributed to the fact that undergraduates set their course goals before they develop a sense of relatedness to their course mates.

The findings on the relationship between relatedness and OSRL extend the previous finding that relatedness is a distinct need from autonomy and competence in online learning settings [41]. On one hand, the role of relatedness differs from the roles of autonomy and competence. On the other hand, there is overlap between the roles of relatedness and autonomy (i.e., enhancing help-seeking) and between the roles of relatedness and competence (i.e., enhancing self-evaluation and task strategies) in promoting OSRL.

From the perspective of OSRL strategies, the study findings can be interpreted as follows. Different OSRL strategies were responsive to the satisfaction of different needs: (a) Resource management strategies, such as time management, environment structuring, and help seeking, were more sensitive to the satisfaction of autonomy than to the satisfaction of competence or relatedness (except for help seeking); (b) four out of six strategies were associated with competence; and (c) no OSRL strategy was associated with all three needs, which underscores the distinct contributions of autonomy, competence, and relatedness to the various aspects of self-regulated learning.

In sum, this study demonstrates the positive association between need satisfaction and OSRL, highlighting the importance of addressing undergraduates’ psychological needs in the context of online learning. This study also determines the specific associations between each need satisfaction and different OSRL strategies, highlighting the unique role of each need in OSRL strategies.

### Implications

The study findings have theoretical and practical implications. Theoretically, the findings confirm the validity of the SDT in OSRL. Although online learning settings are different from face-to-face settings, satisfying the basic psychological needs of autonomy, competence, and relatedness could influence students’ self-regulation in an online-learning environment. In particular, perceived competence has been shown to promote self-regulation in online courses as in face-to-face courses (e.g., [13,14]). The findings also highlight the importance of considering each need satisfaction individually rather than treating need satisfaction as a single construct. Acknowledging the distinct role of each need in influencing different OSRL strategies allows for a more targeted approach to fostering self-regulated learning in online courses. Practically, the findings suggest that online course instructors and designers should first identify the most relevant and desired strategy(ies) in specific online learning situations, which will enable them to prioritize the satisfaction of corresponding psychological needs of the undergraduates in accordance with the desired OSRL strategy(ies). Additionally, it is not recommended to expect that satisfying only one need will lead to an increase in the use of all OSRL strategies.

## Conclusion

The study findings have significant implications in the context of the post-COVID era, where online learning may become more prevalent. With the increasing popularity of online courses, understanding the factors that influence students’ self-regulation in this environment becomes increasingly crucial. This study determines the association between need satisfaction and OSRL among 381 Chinese undergraduates taking online general education courses. The study reveals two key findings: (a) overall, need satisfaction is positively associated with OSRL, and (b) each need plays a unique role in promoting the use of OSRL strategies, and these roles are not fully complementary to each other. The findings contribute to the literature on the SDT in the online learning context and highlight the necessity of considering each need individually when designing pedagogical interventions or learning experiences to improve students’ self-regulated learning. For example, to promote undergraduates’ use of metacognitive strategies, their competence in learning online courses should be enhanced.

The study has several limitations. First, the participants in this study were recruited through convenience sampling at a university in South China. Future research should consider replicating the study with more diverse samples to include different cultural backgrounds and/or educational levels to assess the validity of the results obtained in this study. Second, we used a self-report survey to measure OSRL, which may have affected the objectivity and accuracy of OSRL measurement. Future studies could use objective data sources such as log files to assess students’ OSRL. Third, as the study used a cross-sectional design, the causality between need satisfaction and the use of OSRL strategies could not be confirmed. Although we employed the SDT to hypothesize that fulfilling the three needs leads to an improvement in OSRL strategies, future studies using longitudinal data or experiments can help determine the direction of causality. Moreover, future research could consider validating the findings of this study in blended courses or synchronous online courses.

## Supporting information

S1 Data Participants response.
(XLSX)
